# The effectiveness of goal setting and monitoring in school‐based humanistic counselling with young people: Pilot cluster randomised controlled trial

**DOI:** 10.1111/papt.70094

**Published:** 2026-07-23

**Authors:** Charlie Duncan, David Saxon, Jacqueline Hayes, Mick Cooper

**Affiliations:** ^1^ School of Psychology University of Roehampton London UK; ^2^ Research Department British Association for Counselling and Psychotherapy Lutterworth UK; ^3^ Psychology Department University of Sheffield Sheffield UK; ^4^ Independent Practitioner‐Researcher Lewes UK

**Keywords:** cluster randomised controlled trial, feasibility trial, goals, school‐counselling, young people

## Abstract

**Objective:**

To determine the feasibility and acceptability of a cluster RCT comparing school‐based humanistic counselling (SBHC) plus a goal‐based task (SBHC+G) with SBHC alone. A secondary aim was to establish preliminary indicators of change on measures of psychological distress and life functioning.

**Design:**

This was a pilot cluster randomised controlled trial.

**Method:**

Recruitment and retention of counsellors and young people, as well as measure completion rates and session attendance, were analysed as indicators of acceptability feasibility. ANCOVA tests were used to compare endpoint scores between conditions, controlling for baseline scores, on measures of psychological distress (YP‐CORE) and life functioning (ORS).

**Results:**

Recruitment, retention and measure completion rates were acceptable. There were no statistically significant differences between conditions on YP‐CORE or ORS scores at end point. Those in the SBHC+G condition missed significantly fewer sessions (M = 1.7) than those in the SBHC only condition (M = 3.2, *p* = .04).

**Conclusion:**

The trial design was feasible and acceptable and has the potential to be scaled up to a larger, adequately powered study.

## OBJECTIVE

Schools are commonly utilised settings for young people accessing mental health services, both in the general population and those experiencing more elevated levels of symptoms or clinical diagnoses (Duong et al., 2021). As such, they play a key role in the prevention and treatment of mental health conditions, offering a broad range of therapeutic interventions.

The nature of school‐based mental health provision varies globally. In the United States (US), such interventions take a predominantly cognitive behavioural therapy (CBT) approach (Arora et al., 2019). In contrast, in the United Kingdom (UK), they are more likely to take a humanistic or integrative approach (Copeland et al., 2023). School‐based humanistic counselling (SBHC) in the UK is a manualised intervention, derived from evidence‐based competences for working with young people (BACP, 2022; Cooper et al., 2021). SBHC typically includes techniques such as empathic reflecting, active listening and using creative methods to encourage and support young people to express and articulate their emotions.

A specific evidence‐based competency in SBHC is the ‘ability to establish and agree and therapeutic focus/goals for the counselling’, which also includes ‘an ability to record the child/young person's identified goals for the counselling, with the aim of evaluating whether they have been met by review dates or at the end of the counselling’ (BACP, 2022; p. 88). Whilst integrated within SBHC, goal setting and monitoring is not exclusive to this therapeutic approach. Goal‐oriented practices are also common components of other modalities, including cognitive behavioural therapy (CBT; NHS England, 2024), and have received growing attention within psychotherapy research more broadly (Sales et al., 2023).

Therapeutic goals have been defined as ‘conscious… intentions, desires, and purposes’ (Locke, 1968; p. 158). Within the psychotherapeutic literature, ‘goal‐oriented practices’ typically incorporate three key elements: goal setting (i.e. identifying goals), goal monitoring (i.e. the tracking of progress towards, or away from, goals) and goal discussion (i.e. any collaborative discussion of goals between client and therapist; Jacob et al., 2025). Existing research with adults indicates that goal setting and monitoring, particularly when done publicly (i.e. at least one other person is aware of the goals), and where there is a physical recording of progress, has small‐to‐medium positive effects on the rate of goal attainment (*d =* .40; Harkin et al., 2016) and small positive effects (*d =* .20) on mood and depressive symptoms. Within therapy, clients with more positive attitudes to goal‐oriented practices make significantly greater improvements than those with negative attitudes (Di Malta et al., 2019).

Cairns et al. (2019) found that young people who had set at least one goal at the start of therapy, irrespective of goal quality, were significantly less likely to disengage from therapy and attended significantly more therapeutic sessions compared to those who did not set a goal. Jacob et al. (2017) found that parents of young people attending statutorily funded psychological therapies were more likely to be satisfied with the care they received when the young person had set goals, compared to those who had not. Whilst the literature presented highlights the apparent positive role that goal setting and monitoring can have on therapeutic outcomes, few controlled studies have been conducted which allocate clients to a goal‐oriented versus non‐goal‐oriented intervention. This means causality cannot be established. Lloyd et al. (2019), in their systematic review of idiographic goal‐based measures, concluded that:

an essential area for further research in this field is to examine, in more detail, whether the benefits of goal setting and goal monitoring, as identified in the psychological literature do, indeed, transfer to psychotherapy. Controlled studies, with and without the use of goal measures… would be a robust means of assessing this. (p. 11)

McMurran et al. (2013) conducted a feasibility study for a randomised controlled trial (RCT) with adults clients in a personality disorder service, comparing a goal‐based task plus treatment as usual against treatment as usual alone. The goal‐based task was a 1.5 h motivational interview prior to the start of treatment, with the completion of an idiographic goal measure, the Personal Concerns Inventory (PCI; Cox & Klinger, 2000). Over a 12‐week period, clients allocated to the goal‐based task group attended a median of 83.3% of sessions, whereas those in the treatment as usual group attended a median of 66.7% of sessions, which represents a medium effect size (*d =* 0.44). Clients allocated to the goal‐based task group also scored significantly higher on a rating of treatment engagement (i.e. were more engaged in treatment) than those in the treatment as usual group, which represented a very large effect size (*d* = 1.62). It should be noted that the motivational interview task was undertaken by a researcher, rather than the practitioner providing the intervention, and therefore the extent to which practitioners were aware of the goals that clients set, and the extent to which goal monitoring was embedded into the intervention, cannot be determined.

To date, there are considerable gaps in our understanding and a lack of robust methodology to adequately determine the extent to which goal‐oriented practices lead to significant improvements in outcomes for young people. The primary aim of the present study, therefore, was to determine the feasibility and acceptability of a cluster RCT to assess the comparative effectiveness of an intervention, school‐based humanistic counselling, with and without an integrated goal‐based task (SBHC+G vs. SBHC, respectively). This was assessed through a range of factors, including: recruitment, retention, session attendance and measure completion rates, identifying any significant differences in these variables between the two conditions. The secondary aim was to establish preliminary outcomes on measures of psychological distress and life functioning to power a main trial.

## METHOD

### Design

In this pilot cluster RCT design, counsellors were randomised to either a school‐based humanistic counselling plus goals (SBHC+G) condition or SBHC alone. Randomisation was clustered at the counsellor level, rather than an individualised RCT. This decision was made to limit the potential for contamination from the SBHC+G to the SBHC‐only condition by counsellors inadvertently introducing formal goal setting and monitoring processes with control clients. Due to differences in the caseloads of counsellors, the number of young people in each condition was not equal.

Young people in both conditions received up to 10 sessions of counselling over 10 school weeks, with all asked by their counsellor to complete a measure of life functioning at each session, and a measure of psychological distress at baseline and at their last planned session. Young people in the SBHC+G condition were also asked by their counsellor to set up to three personal goals in their initial sessions and rate their progression towards them at each session.

### Procedure

The present cluster RCT was undertaken as a follow‐up to a fully powered individualised RCT of SBHC and pastoral care as usual compared to pastoral care alone (PCAU; for the trial protocol see Stafford et al., 2018), which took place across 18 secondary schools in London, UK. To be eligible for inclusion in the initial RCT, young people had to: be 13–16 years of age; be experiencing moderate to severe levels of psychological distress as assessed by a score of 5 or more on the Strengths and Difficulties Questionnaire Emotional Symptoms (SDQ‐ES) scale (Goodman, 2001); have an English reading age of 13 or above; want to take part in counselling and not be in receipt of another counselling intervention; and have a school attendance record of at least 85% (Stafford et al., 2018).

Young people randomised to the PCAU condition in the initial RCT were offered the SBHC intervention following the 24‐week follow‐up testing period. This presented an additional opportunity to undertake research to explore the feasibility and acceptability of a cluster RCT comparing SBHC+G with SBHC only.

All counsellors in the initial RCT (*n* = 19) were offered the opportunity to participate in the present study via an email from the first author. The email stated that the aim of the study was to ‘look at the impact that setting and monitoring goals on a session‐by‐session basis may have on the outcomes of counselling (as compared to not)’.

### Consent

Counsellors were asked via email to provide their written consent to participate in the present study. Young people and their parent/carer had already provided written consent to participate in counselling and the research as part of the initial RCT and therefore their additional written consent was not required for the present study. This process received ethical clearance through the University of Roehampton Research Ethics Committee (REF: PSYC 17/262).

### Participants

Ten of the 19 counsellors (52.6%) provided written consent to be included in the present study and were randomly assigned (1:1) using an online random number generator to the SBHC+G condition (*n* = 5) or SBHC only condition (*n* = 5). Of the counsellors included in the present study, the majority were female (*n* = 7, 70%), of white ethnicity (*n* = 7, 70%) and not disabled (*n* = 10, 100%), which was not observably different from the demographic profile of counsellors in the initial trial. Each counsellor worked in a different school. Hence, 10 schools were included in the present study.

A total of 91 young people (across the 10 counsellors who consented to participate) aged 13–16 from the initial trial were available to participate in the present study. Sixteen (17.6%) did not take up the offer of counselling following the initial trial and therefore were not included in the present study. There were no observable differences in the demographic profile of young people who did not take up the offer of counselling following the initial trial and those who did in terms of gender, school year, ethnicity or disability status. A further seven young people (7.7%) had started counselling prior to the ethical procedures for the present study being approved and therefore were not included. Of the remaining 68 young people (74.7% of the available sample), 41 (60.3%) were seen by counsellors randomised to the SBHC+G condition (experimental) and 27 (39.7%) were seen by counsellors randomised to the SBHC only (control) condition. Figure 1 presents a flowchart of the participants to the cluster RCT.

**FIGURE 1:**
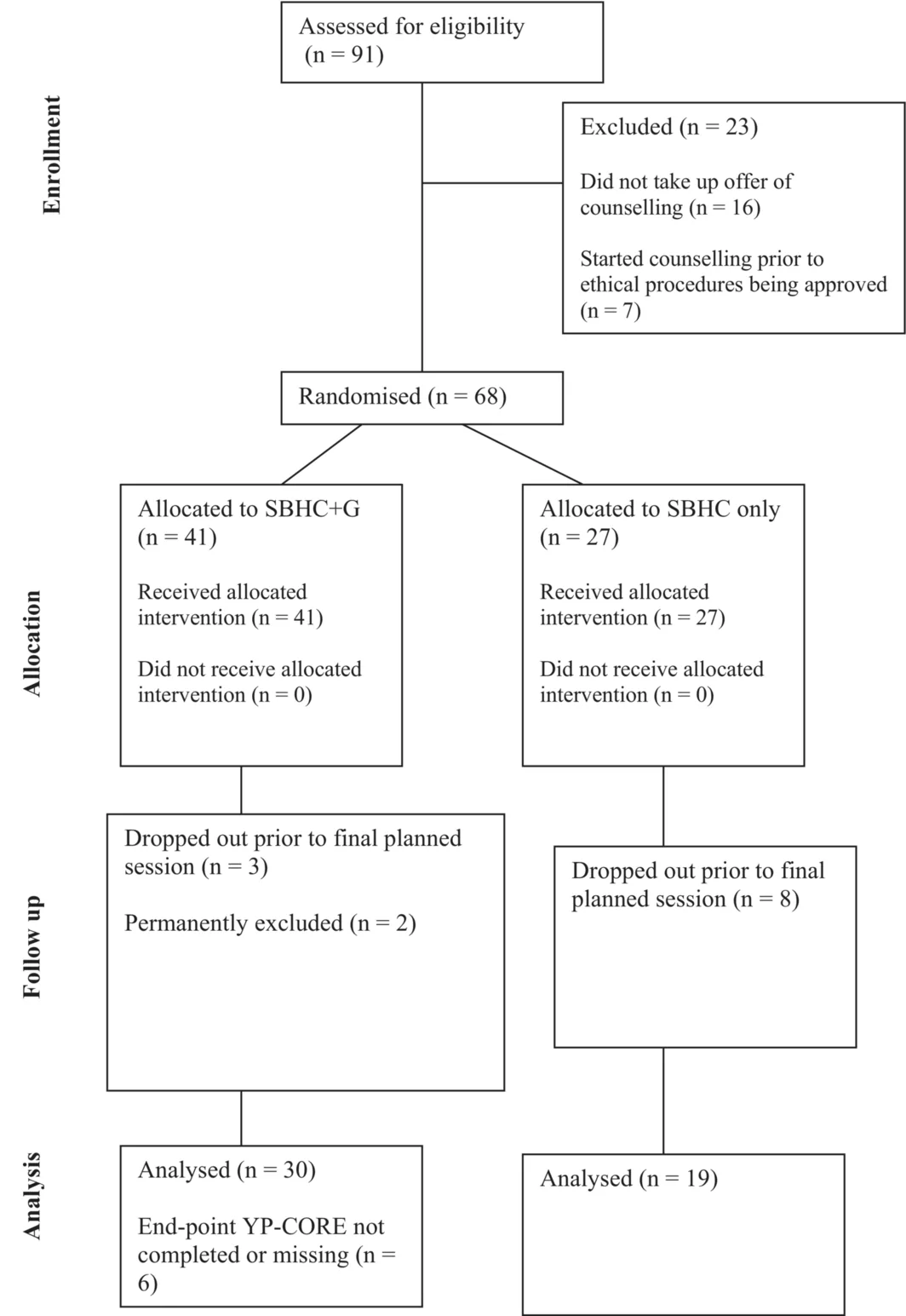
Flow chart of RCT participants.

An overview of the demographic profile of the young people assigned to each condition is presented in Table 1. There were no observable differences in the demographic profiles of young people assigned to each condition in terms of gender (*χ* ^2^ (2,68) = .93, *p* = .63), ethnicity (*χ* ^2^ (1,67) = 1.82, *p* = .18) or age (*t*(62.05) = −.95, *p* = .34).

**TABLE 1:** Demographic profile of young people by condition.

Demographic variable	SBHC+G condition, *n* (%^a^)	SBHC only condition, *n* (%^a^)
Gender		
Female	37 (90.2)	24 (88.9)
Male	3 (7.3)	3 (11.1)
Other	1 (2.4)	0 (0.0)
Age (mean, SD)	13.7 (0.8)	13.9 (0.7)
Ethnicity		
White, European and/or British	20 (48.8)	18 (66.7)
Black, mixed or other	20 (48.8)	9 (33.3)
Missing	1 (2.4)	0 (0.0)

^a^ Percentages calculated as a proportion of each condition i.e. of the 41 young people in the SBHC+G condition, 37 (90.2%) were female.

### Intervention

All counsellors randomised to the SBHC+G condition were provided with a half day training on the cluster RCT protocol and the setting and monitoring of goals using the Goal Based Outcome (GBO) tool (Law, 2011; 2019). They were also provided with access to online learning materials in the form of the GBO manual (Law & Jacob, 2013) and an e‐learning module on ‘establishing a therapeutic goal’ (Law, 2016).

Young people in the SBHC+G were invited by their counsellor to set up to three personal goals for counselling during their initial sessions using the GBO tool (Law, 2011) and rate their progress on each goal at each counselling session thereafter.

SBHC is a manualised form of counselling (Kirkbride et al., 2016) based on humanistic principles and competences developed for working with children and young people (BACP, 2022). The principles and philosophy that underpin this approach focus on the role of the therapist in ‘[providing] conditions in the therapeutic relationship that will allow the actualisation [the innate desire to fulfil one's own potential] process to the facilitated’ (Kirkbride et al., 2016; p.8). These are the conditions of congruence, unconditional positive regard and empathy. This may include using skills such as ‘active listening’ and ‘empathic reflections’ and supporting the young person to make sense of their own experiences and behaviours.

Young people in both conditions were invited to complete a measure of life functioning—the Outcome Rating Scale (ORS; Miller et al., 2003)—at every session by their counsellor. All young people were invited to complete a measure of psychological distress—the Young Person's Clinical Outcomes in Routine Evaluation (YP‐CORE; Twigg et al., 2009)—at baseline (their follow‐up testing point for the fully‐powered RCT, just prior to participation in the present study) and endpoint (their last, planned therapeutic session) by their counsellor. Counsellors were encouraged to integrate the measures into the therapeutic work, but there was no mandate for when they should be completed during a session.

### Measures

#### Goal based outcome (GBO) tool

The GBO tool (Law, 2011) allows up to three personal goals to be recorded for counselling using a free‐text box. These goals can be set by young people, parents/carers, or practitioners, or a combination of all three. For the purposes of the current study, all goals were set by the young people themselves. Each goal is written at the top of a separate sheet of paper, either by the young person or counsellor. Below the written goal are 12 rows, with a column on each row where the session date can be recorded. Each row contains the numbers zero (‘not met at all’) to ten (‘fully met’), which can then be circled by the young person or counsellor to indicate their progress on each individual goal.

The GBO tool has demonstrated moderate levels of test–retest stability over the medium term (6–12 weeks), as well as moderate convergence with some other measures of psychological wellbeing (Duncan et al., 2023). A reliable change index of 3 points has been calculated previously for the GBO tool (Duncan et al., 2023; Edbrooke‐Childs et al., 2015). Where a young person sets multiple goals, degrees of reliable change can be categorised in four ways: *reliable change* (reliable change on at least one goal, with no reliable deterioration on any single goal), *reliable deterioration* (reliable deterioration on at least one goal, with no reliable improvement on any single goal), *no reliable change on any goals* and *inconclusive* (reliable change on at least one goal and reliable deterioration on at least one goal; Jacob et al., 2021).

#### Outcome rating scale (ORS)

The ORS (Miller et al., 2003) is a four‐item self‐report visual analogue scale which measures four domains of life functioning: individual (personal well‐being), interpersonal (close relationships and relationships with family), social (work, school and friendships) and overall (general sense of wellbeing). Each item is scored from 0–10 by making a mark on a 10 cm line indicating ‘how well they have been doing over the past week’, with marks to the left indicative of lower levels of functioning and marks to the right being indicative of higher levels of functioning. Total scores range from 0 to 40 and a clinical cut‐off of 28 has been established for young people aged 13–17 years. Scores of 28 and below are considered to be within the clinical range. The ORS has been shown to have good levels of reliability and validity (Miller et al., 2003) and a reliable change index (RCI) of 6 points has been calculated previously (Redmayne et al., 2024). Using data from the present study, an RCI of 5.48 was calculated, which is in line with previous calculations. Hence, a change of 6 or more points in either direction was considered reliable (i.e. not a result of measurement error).

#### Young Person's Clinical Outcomes in Routine Evaluation (YP‐CORE)

The YP‐CORE (Twigg et al., 2009) is a ten‐item self‐report measure of psychological distress, validated for use with 11–17 year olds. Each item is scored between 0 (‘not at all’) to 4 (‘most or all of the time’), resulting in total scores ranging from 0 to 40, with higher scores being indicative of higher levels of psychological distress. It asks young people how they have felt over the past week in response to statements such as ‘my thoughts and feelings distress me’ and ‘I've felt unhappy’. YP‐CORE has previously demonstrated good levels of reliability and validity and has established age‐ and gender‐specific reliable and clinical change indices (see Twigg et al., 2016).

### Analytic strategy

Indicators of feasibility and acceptability—counsellor recruitment, counsellor and young person retention and measure completion rates—were assessed using descriptive statistics. Independent samples *t*‐tests and chi‐squared or Fisher's exact tests were undertaken to determine whether there were any statistically significant differences in these variables by condition, where appropriate. The following targets, based on a previous trial of SBHC (Cooper et al., 2021) and progression criteria set for publicly funded RCTs (Herbert et al., 2019), were set to determine the feasibility and acceptability of the intervention:

- 50% of eligible counsellors recruited.
- 80% counsellor retention
- 80% young person retention
- 75% measure completion rates
- Average of 6 sessions attended

To establish preliminary indications of effectiveness, session attendance, baseline scores on outcome measures and baseline to endpoint reliable and clinical change were analysed descriptively. For reliable and clinical change, the following baseline to endpoint categories were compared on GBO tool, YP‐CORE and ORS: (a) reliable and clinical improvement versus all other change categories (b) reliable improvement (with or without clinical improvement) versus all other change categories (c) clinical improvement (with or without reliable improvement) versus all other clinical change categories and (d) reliable deterioration versus all other categories.

Independent samples *t*‐tests and chi‐squared, or Fisher's exact tests, were then undertaken to determine any statistically significant differences between conditions on YP‐CORE and ORS, as the GBO tool was not completed in the SBHC‐only condition. Effect sizes (ESs) were reported using *Cohen's d* or Glass's Δ (where there were unequal variances), with ESs of 0.2, 0.5, and 0.8 representing small, moderate, and large effect sizes, respectively (Cohen, 1988). Session attendance was categorised as either ‘attended’ or ‘did not attend (DNA)’ (with or without giving prior notice).

Given the hierarchical structure of the data (participants nested within counsellors), analyses were undertaken to determine the necessity of multilevel modelling. As all counsellors, apart from one who worked across two of the ten schools, each worked at a different school, it was assumed that school‐level variance would be observable at the counsellor level. A null model determined that the variance at the counsellor level was non‐significant (ICC = 0.00 [95% CI: [0.00, 0.00], *p* = 1.00]) and therefore multilevel modelling was deemed unnecessary. Hence, differences between mean endpoint scores on the ORS and YP‐CORE between conditions were analysed using analysis of covariance (ANCOVA), controlling for baseline scores. As the ORS was used at each session, where a participant discontinued counselling before their final planned session, the ORS score from their last attended session was used as the endpoint score.

Statistical analyses were undertaken using the Statistical Package for the Social Sciences (SPSS) version 29 (IBM Corp, 2023) and MLwiN 3.08 (Centre for Multilevel Modelling, 2023).

## RESULTS

### Recruitment and retention

Ten of the nineteen counsellors recruited to the initial trial (52.6%) provided consent to be included in the cluster RCT. Four (21.1%) counsellors did not respond to the request to participate in the present research, three (15.8%) declined to participate due to not wanting to introduce additional outcome measures to their work, one (5.3%) declined due to workload, and one (5.3%) indicated that they would be willing to participate but did not provide written consent. One counsellor out of the ten recruited (10%) dropped out of the study prior to completion due to finding permanent work. However, another counsellor who had already been recruited to the study and who had been randomised to the same condition took on their final cohort of young people.

Of the 68 young people recruited to the trial, a total of 13 (19.1%) dropped out of the study: two (2.9%) of whom were permanently excluded from school whilst participating in the study and 11 (16.2%) who finished counselling before their final planned session. Of those who dropped out of counselling, five had been randomised to the SBHC+G condition and eight had been randomised to the SBHC only condition, which represents 12.2% and 29.6% of young people randomised to each condition, respectively. This did not represent a statistically significant difference in retention rates between the two conditions, although it was approaching significance (*χ* ^2^ (1,68) = 3.20, *p* = .07).

### Measure completion rates

Table 2 presents an overview of endpoint measures' completion rates, both overall and by each condition where appropriate. Endpoint measures' completion rates ranged from 65.9% to 85.2%. There were no statistically significant differences between conditions in terms of completion rates on ORS (*χ* ^2^ (1,68) = .91, *p* = .34) or YP‐CORE (*χ* ^2^ (1,68) = .06, *p* = .80). Endpoint scores on all measures were missing from six young people, all in the SBHC+G condition, as schools reported misplacing the data. There were a further 15 young people across both conditions (22.1%) who were missing endpoint scores on at least one measure; seven of these (46.7%) were from the SBHC+G condition and eight (53.3%) from the SBHC only condition. Six of the 15 (40.0%) young people for whom at least one endpoint measure was missing—and which could not be attributed to schools misplacing data—completed the study: three from the SBHC+G group and three from the SBHC only group. This represents 8.8% of the total sample of 68 young people.

**TABLE 2:** Endpoint measures' completion rates.

Measure	SBHC+G, *n* = 41	SBHC, only *n* = 27	Overall, *n* = 68
GBO	27 (65.9%)	N/A	N/A
YP‐CORE	30 (73.2%)	19 (70.4%)	49 (72.1%)
ORS	31 (75.6%)	23 (85.2%)	54 (79.4%)
GBO Tool	29 (70.7%)	N/A	N/A

*Note*: Percentages calculated as a proportion of each condition i.e. of the 41 young people in the SBHC+G condition, 30 (73.2%) completed YP‐CORE at endpoint.

### Session attendance

Across both conditions, young people attended an average of 7.6 (SD = 2.66) sessions (range 1–10) and DNA 2.3 (SD = 2.67) sessions (range 0–9). In the SBHC+G condition, young people attended an average of 8.1 (SD = 2.20) sessions compared to 6.8 (SD = 3.13) in the SBHC only condition. This did not represent a statistically significant difference, albeit it was approaching significance (*t*(42.77) = 1.85, *p* = .07). However, there was a statistically significant difference in the number of DNA sessions in each condition, with those in the SBHC only condition not attending significantly more sessions (*M* = 3.2, SD = 3.19) than those in the SBHC+G condition (*M* = 1.7, SD = 2.16, *t*(42.2) = −2.18, *p* = .04). This was approaching a moderate effect size (Glass's Δ = .48).

### Measure scores

Table 3 presents an overview of baseline and endpoint GBO, YP‐CORE, and ORS scores, both overall and by each condition, where appropriate. There were no statistically significant differences between baseline YP‐CORE scores (*t*(66) = .90, *p* = .37) or baseline ORS scores (*t*(55) = −1.47, *p* = .15) in the two conditions.

**TABLE 3:** Baseline and endpoint scores.

Measure	SBHC+G, *M* (SD)	SBHC only, *M* (SD)	Overall, *M* (SD)
GBO (baseline)	2.8 (1.67)	N/A	N/A
GBO (endpoint)	5.7 (2.37)	N/A	N/A
YP‐CORE (baseline)	19.6 (7.5)	18.1 (6.3)	19.0 (7.0)
YP‐CORE (endpoint)	17.4 (7.6)	15.9 (7.5)	16.8 (7.5)
ORS (baseline)	20.2 (7.7)	23.1 (7.0)	21.5 (7.5)
ORS (endpoint)	24.3 (9.5)	25.4 (7.8)	24.8 (8.8)

### Endpoint YP‐CORE scores

Prior to controlling for baseline scores, the mean endpoint YP‐CORE score for the SBHC+G group was 17.4 (SD = 7.59) and for the SBHC only group it was 15.9 (SD = 7.46). After controlling for baseline YP‐CORE scores, the adjusted mean for endpoint YP‐CORE scores in the SBHC+G group was 16.4 and for the SBHC only group it was 17.4. Baseline YP‐CORE scores were a significant predictor of endpoint YP‐CORE scores, *F*(1,46) = 30.1, *p* < .001, partial *η* ^2^ = .40. Condition did not have a significant effect on endpoint YP‐CORE scores after controlling for baseline YP‐CORE scores, *F*(1,46) = .26, *p* = .61.

### Endpoint ORS scores

Prior to controlling for baseline scores, the mean endpoint ORS score for the SBHC+G group was 24.3 (SD = 9.5) and for the SBHC only group it was 25.4 (SD = 7.8). After controlling for baseline ORS scores, the adjusted mean for endpoint ORS scores in the SBHC+G group was 24.9 and for the SBHC only group it was 24.5. Baseline ORS scores were a significant predictor of endpoint ORS scores, *F*(1,51) = 17.0, *p* < .001, partial *η* ^2^ = .25. Condition did not have a significant effect on endpoint ORS scores after controlling for baseline ORS scores, *F*(1,51) = 0.04, *p* = .84.

### Reliable and clinical change

Table 4 presents an overview of baseline to endpoint reliable and clinical change on GBO, YP‐CORE and ORS, both overall and by each condition, where appropriate.

**TABLE 4:** Reliable and clinical change.

Measure	SBHC+G, *n* (%)	SBHC only, *n* (%)	Total, *n* (%)
GBO			
Reliable improvement	18 (62.1%)	N/A	N/A
Reliable deterioration	0 (0%)	N/A	N/A
No reliable change	11 (37.9%)	N/A	N/A
Inconclusive	0 (0%)	N/A	N/A
YP‐CORE			
Reliable improvement	11 (36.7%)	3 (15.8%)	14 (28.6%)
Reliable deterioration	0 (0%)	0 (0%)	0 (0%)
No reliable change	19 (63.3%)	16 (84.2%)	35 (71.4%)
Reliable and clinical improvement	7 (23.3%)	1 (5.3%)	8 (16.3%)
ORS			
Reliable improvement	13 (41.9%)	7 (30.4%)	20 (37.0%)
Reliable deterioration	5 (16.1%)	1 (4.3%)	6 (11.1%)
No reliable change	13 (41.9%)	15 (65.2%)	28 (51.9%)
Reliable and clinical improvement	8 (25.8%)	2 (8.7%)	10 (18.5%)

Across both conditions, 14 of the 49 participants who had endpoint YP‐CORE scores showed reliable improvement (28.6%), 0 (0%) showed reliable deterioration, and 35 (71.4%) showed no reliable change. Of those who made reliable improvement, eight (16.3%) also showed clinical improvement. In the SBHC+G group, for whom data was available from 30 participants, 11 (36.7%) showed reliable improvement on YP‐CORE and the remaining 19 (63.3%) showed no reliable change. Of those who made reliable improvement, 7 (23.3%) also showed clinical improvement. In the SBHC only group, for whom data was available from 19 participants, these figures were three (15.8%), 16 (84.2%) and two (8.7%), respectively. Fisher's exact tests showed that there were no statistically significant differences between conditions in the proportions of young people showing reliable and clinical improvement (*p* = .13), clinical improvement (with or without reliable improvement; *p* = .32) or reliable improvement (with or without clinical improvement; *p* = .19) on YP‐CORE. As no young people showed reliable deterioration on YP‐CORE, it was not possible to assess for differences between conditions on this change category.

Across both conditions, 20 of the 54 participants who had endpoint ORS scores showed reliable improvement (37.0%), six (11.1%) showed reliable deterioration and 28 (51.9%) showed no reliable change. Of those who made reliable improvement, 10 (18.5%) also showed clinical improvement. In the SBHC+G group, for whom data was available from 31 participants, 13 (41.9%) showed reliable improvement on ORS, five (16.1%) showed reliable deterioration, and 13 (41.9%) showed no reliable change. Of those who made reliable improvement, eight (25.8%) also showed clinical improvement. In the SBHC only group, for whom data was available from 23 participants, these figures were seven (30.4%), one (4.3%), 15 (65.2%) and two (8.7%), respectively. Fisher's exact tests showed that there were no statistically significant differences between conditions in the proportions of young people showing reliable and clinical improvement (*p* = .16), clinical improvement (with or without reliable improvement; p = .32), reliable improvement (with or without clinical improvement; *p* = .41) or reliable deterioration (*p* = .22) on ORS.

## DISCUSSION

Findings from the present study indicate moderate overall feasibility and acceptability of the trial design, measures and intervention for both counsellors and young people. Whilst some counsellors provided informal indications that they were reluctant to participate due to perceived workload and additional outcome measures, engagement among young people was strong, with over 80% retention and 70% measure completion rates. Whilst the measure completion rates were slightly lower than the 75% target, particularly for the GBO tool, the relatively high displacement of data by schools (~10%) may provide a partial explanation for this. This is not uncommon in complex settings like schools, but measures taken to address this in future research should be considered. One potential solution to mitigate this risk in future studies could be to utilise electronic means of data collection, rather than paper copies, such as the use of tablets in session. Another recommendation would be to gather qualitative feedback from counsellors and young people about the acceptability of the outcome measures. The inclusion of goal setting and monitoring (SBHC+G) appeared to enhance session attendance and engagement compared to SBHC alone, supporting the feasibility of a larger, fully powered trial to further investigate the impact on outcomes.

Recruitment to the present study was satisfactory, with over half of the counsellors from the larger RCT agreeing to participate, which was in line with the pre‐defined criteria set. A smaller proportion chose not to take part, primarily citing concerns about additional workload or the introduction of further outcome measures; however, other counsellors did not respond to the call to participate. It is possible then that with more encouragement and follow‐up, more counsellors may have consented to participate. In addition, given that the perceived burden of using an additional outcome measure was unappealing for some counsellors, more could be done in future studies to engage counsellors in the process, such as offering training in using the measures prior to the invitation to participate to alleviate potential concerns. Furthermore, considerations should be given to the location of the present trial. All counsellors were based in secondary schools in London, which provided greater opportunities for in‐person training and supervision, perhaps enhancing peer support opportunities between counsellors which may not have been possible had the trial taken place over a larger geographical area. This may have implications for recruitment and retention rates in any subsequent trials.

More encouraging, perhaps, was the recruitment of young people where only 17.6% of those eligible to participate did not take up the offer of counselling. This is, however, probably unsurprising given that they had previously consented to participate in the large scale RCT and were assigned to the waiting list. Nevertheless, it is encouraging that over 80% of young people were eligible and willing to be included. Similarly, retention to the trial was good, with over 80% of young people and 90% of counsellors seeing it through to completion. Young people across both conditions attended an average of 7.6 sessions, which was greater than the average of 6 sessions defined as an indicator of intervention acceptability. Taken together, these findings suggest that the trial design, measures, and the intervention are acceptable to both counsellors and young people. It is, however, important to note that, in addition to some counsellors being discouraged from participating, some clients (8.8%) completed the intervention but did not complete some or all of the outcome measures. Reasons for this are unclear and it is a limitation of the present study that mechanisms were not in place to investigate this further.

Our data identified a trend approaching statistical significance for those in the SBHC+G condition to attend more sessions than those in the SBHC only group, which would align with findings from Cairns et al. (2019). Indeed, a statistically significant difference was found in the number of DNA sessions, with those in the SBHC only condition not attending significantly more sessions than those in the SBHC+G group. One possible explanation for these findings is that the process of goal setting and monitoring increases motivation and accountability—and hence engagement—over and above that for young people receiving SBHC only, which has been suggested previously (Tryon, 2018). Another, albeit linked, tentative explanation which has been proposed previously (Jacob et al., 2022) is that the process of goal setting between a young person and their counsellor contributes to a good alliance, by building a sense of trust and open communication, which in turn can lead to greater session attendance (Ryan et al., 2023).

Differences in outcomes on measures of psychological distress and life functioning between the two conditions were not statistically significant. This may be particularly pertinent given that both conditions were active interventions and there was no non‐intervention control group. Hence, any differences between conditions are likely to be of a smaller magnitude. Nevertheless, there is no indication that the inclusion of goal setting and monitoring in SBHC is associated with poorer outcomes compared to SBHC alone. It should also be noted that standardised measures such as YP‐CORE and ORS tend to be less sensitive to individual change than idiographic measures, such as the GBO tool. It is possible, therefore, that differences in change on other idiographic measures may be more pronounced between conditions and may be considered more appropriate for trials where there is an active control group. Future studies that are powered and designed to detect meaningful differences may be warranted given the feasibility and acceptability of the present trial design and intervention.

In terms of further limitations of our study, whilst SBHC+G counsellors were provided with training, it was limited to half a day, and there was no recording of sessions nor measure of adherence to the goal setting and monitoring processes. Therefore, it is likely that there were differences in how counsellors implemented and integrated goal setting and monitoring. This aligns with the conclusions of Jacob et al. (2025) that practice principles and fidelity tools to assess adherence to a goal‐oriented approach need to be developed and trialled. Similarly, there was no measure of adherence to the SBHC intervention itself in the present study, which is also considered an indicator of intervention feasibility. However, the counsellors included in the present study were recruited from a larger trial where their adherence to the SBHC intervention was considered acceptable (Cooper et al., 2021).

Second, we did not explicitly gather feedback from counsellors and young people on the trial protocol and measures used. Rather, we determined feasibility and acceptability using proxy measures such as retention and measure completion rates. Whilst qualitative research has been published which explored young people's experiences of the goal setting and monitoring process in SBHC (Duncan et al., 2025), it did not seek to compare differences in experiences between young people assigned to an SBHC+G group compared to SBHC only, which may provide additional information about the acceptability and impact of goal‐oriented practice. Future research should look to qualitative methods to gather feedback more explicitly to inform trial design and impact beyond that captured using outcome measures. Finally, due to randomisation occurring at the counsellor level, more young people were randomised to the SBHC+G group than the SBHC only group. This could have decreased the power of the present study and increased the likelihood of Type I errors (Murray et al., 2004).

In conclusion, the present study demonstrates that a fully powered cluster RCT of SBHC+G vs. SBHC alone would be feasible. This would allow us to determine the extent to which, if at all, the addition of goal setting and monitoring, in comparison to SBHC alone, improves outcomes. Future research should also look to develop best practice principles for working with goals, along with a fidelity tool to assess adherence. In addition, the collection of qualitative data would allow for a greater exploration of outcomes and the acceptability of such a trial design to young people and counsellors.

## AUTHOR CONTRIBUTIONS

**Charlie Duncan:** Conceptualization; investigation; funding acquisition; writing – original draft; methodology; writing – review and editing; formal analysis; project administration; data curation. **David Saxon:** Conceptualization; methodology; writing – review and editing; supervision. **Jacqueline Hayes:** Conceptualization; supervision; writing – review and editing. **Mick Cooper:** Conceptualization; funding acquisition; methodology; writing – review and editing; supervision.

## CONFLICT OF INTEREST STATEMENT

CD, DS and MC received funding from the Economic and Social Research Council (grant reference ES/M011933/1). CD is employed as a researcher at the British Association for Counselling and Psychotherapy.

## ETHICS STATEMENT

This study received ethical clearance through the University of Roehampton Research Ethics Committee (REF: PSYC 17/262).

## Data Availability

The data that support the findings of this study are available from the corresponding author upon reasonable request.

## References

[papt70094-bib-0001] Arora, P. G. , Collins, T. A. , Dart, E. H. , Hernández, S. , Fetterman, H. , & Doll, B. (2019). Multi‐tiered systems of support for school‐based mental health: A systematic review of depression interventions. School Mental Health, 11, 240–264. 10.1007/s12310-019-09314-4

[papt70094-bib-0002] BACP . (2022). Competences for work with children and young people (4–18 years). https://www.bacp.co.uk/media/15873/bacp‐cyp‐competence‐framework_2022.pdf

[papt70094-bib-0003] Cairns, A. J. , Kavanagh, D. J. , Dark, F. , & McPhail, S. M. (2019). Goal setting improves retention in youth mental health: A cross‐sectional analysis. Child and Adolescent Psychiatry and Mental Health, 13(31), 1–8. 10.1186/s13034-019-0288-x PMC6615268 31320924

[papt70094-bib-0004] Centre for Multilevel Modelling . (2023). MLwiN 3.08. University of Bristol.

[papt70094-bib-0005] Cohen, J. (1988). Statistical power analysis for the behavioral sciences (2nd ed.). Lawrence Erlbaum Associates.

[papt70094-bib-0007] Cooper, M. , Stafford, M. R. , Saxon, D. , Beecham, J. , Bonin, E.‐M. , Barkham, M. , Bower, P. , Cromarty, K. , Duncan, C. , Pearce, P. , Rameswari, T. , & Ryan, G. (2021). Humanistic counselling plus pastoral care as usual versus pastoral care as usual for the treatment of psychological distress in adolescents in UK state schools (ETHOS): A randomised controlled trial. The Lancet Child & Adolescent Health, 5(3), 178–189. 10.1016/s2352-4642(20)30363-1 PMC9765869 33484658

[papt70094-bib-0008] Copeland, L. , Willis, S. , Hewitt, G. , Edwards, A. , Jones, S. , Page, N. , Murphy, S. , & Evans, R. (2023). School‐ and community‐based counselling services for children and young people aged 7‐18 in the UK: A rapid review of effectiveness, implementation and acceptability. Counselling and Psychotherapy Research, 24(2), 419–458. 10.1002/capr.12688

[papt70094-bib-0009] Cox, W. M. , & Klinger, E. (2000). Personal Concerns Inventory. Unpublished questionnaire. Bangor University.

[papt70094-bib-0010] Di Malta, G. , Oddli, H. W. , & Cooper, M. (2019). From intention to action: A mixed methods study of clients' experiences of goal‐oriented practices. Journal of Clinical Psychology, 75(10), 1770–1789. 10.1002/jclp.22821 31286525

[papt70094-bib-0011] Duncan, C. , Cooper, M. , & Saxon, D. (2023). Test‐retest stability, convergent validity, and sensitivity to change for the goal‐based outcome tool for adolescents: Analysis of data from a randomised controlled trial. Journal of Clinical Psychology, 79(3), 683–696. 10.1002/jclp.23422 PMC10087879 35975697

[papt70094-bib-0012] Duncan, C. , Hayes, J. , & Cooper, M. (2025). Young people's experiences of setting and monitoring goals in school‐based counselling: A thematic analysis. Psychology and Psychotherapy: Theory, Research and Practice, 98(3), 663–682. 10.1111/papt.12581 PMC12346242 39991810

[papt70094-bib-0013] Duong, M. T. , Bruns, E. J. , Lee, K. , Cox, S. , Coifman, J. , Mayworm, A. , & Lyon, A. R. (2021). Rates of mental health service utilization by children and adolescents in schools and other common service settings: A systematic review and meta‐analysis. Administration and Policy in Mental Health and Mental Health Services Research, 48(3), 420–439. 10.1007/s10488-020-01080-9 32940884

[papt70094-bib-0014] Edbrooke‐Childs, J. , Jacob, J. , Law, D. , Deighton, J. , & Wolpert, M. R. (2015). Interpreting standardized and idiographic outcome measures in CAMHS: What does change mean and how does it relate to functioning and experience? Child and Adolescent Mental Health, 20(3), 142–148. 10.1111/camh.12107 32680398

[papt70094-bib-0015] Goodman, R. (2001). Psychometric properties of the strengths and difficulties questionnaire. Journal of the American Academy of Child & Adolescent Psychiatry, 40(11), 1337–1345. 10.1097/00004583-200111000-00015 11699809

[papt70094-bib-0016] Harkin, B. , Webb, T. L. , Chang, B. P. I. , Prestwich, A. , Conner, M. , Kellar, I. , Benn, Y. , & Sheeran, P. (2016). Does monitoring goal progress promote goal attainment? A meta‐analysis of the experimental evidence. Psychological Bulletin, 142(2), 198–229. 10.1037/bul0000025 26479070

[papt70094-bib-0017] Herbert, E. , Julious, S. A. , & Goodacre, S. (2019). Progression criteria in trials with an internal pilot: An audit of publicly funded randomised controlled trials. Trials, 20(1), 493. 10.1186/s13063-019-3578-y PMC6688224 31399148

[papt70094-bib-0018] IBM Corp . (2023). IBM SPSS Statistics for Windows, Version 29.0. Released 2023. IBM Corp.

[papt70094-bib-0019] Jacob, J. , da Silva, L. C. , Sefi, A. , & Edbrooke‐Childs, J. (2021). Online counselling and goal achievement: Exploring meaningful change and the types of goals progressed by young people. Counselling and Psychotherapy Research, 21(3), 502–513. 10.1002/capr.12363

[papt70094-bib-0020] Jacob, J. , De Francesco, D. , Deighton, J. , Law, D. , Wolpert, M. , & Edbrooke‐Childs, J. (2017). Goal formulation and tracking in child mental health settings: When is it more likely and is it associated with satisfaction with care? European Child & Adolescent Psychiatry, 26, 759–770. 10.1007/s00787-016-0938-y PMC5489638 28097428

[papt70094-bib-0042] Jacob, J. , Stankovic, M. , Spuerck, I. , & Shokraneh, F. (2022). Goal setting with young people for anxiety and depression: What works for whom in therapeutic relationships? A literature review and insight analysis. BMC Psychology, 10, 171. 10.1186/s40359-022-00879-5 PMC9281142 35831897

[papt70094-bib-0021] Jacob, J. , Wozney, L. , Weie Oddli, H. , Duncan, C. , Chorney, J. , Emberly, D. , Law, D. , Clark, S. , Heien, S. , Boulos, L. , & Cooper, M. (2025). Goal‐oriented practices in youth mental health and wellbeing settings: A scoping review and thematic analysis of empirical evidence. Psychology and Psychotherapy: Theory, Research and Practice, 98(2), 431–477. 10.1111/papt.12564 PMC12065060 39670986

[papt70094-bib-0022] Kirkbride, R. , McGinnis, S. , & Cooper, M. (2016). Effectiveness and Cost Effectiveness Trial of Humanistic Counselling in Schools (ETHOS): clinical practice manual. https://www.roehampton.ac.uk/globalassets/documents/research‐centres/crest/ethos‐clinical‐practice‐manual‐final‐2016‐11‐18.pdf

[papt70094-bib-0023] Law, D. (2011). Goals and goal based outcomes (GBOs): Some useful information. CAMHS Press.

[papt70094-bib-0024] Law, D. (2016). Establishing a therapeutic goal. https://www.minded.org.uk/Component/Details/447268

[papt70094-bib-0025] Law, D. , & Jacob, J. (2013). Goals and goal based outcomes (GBOs): Some useful information. CAMHS Press.

[papt70094-bib-0026] Lloyd, C. E. M. , Duncan, C. , & Cooper, M. (2019). Goal measures for psychotherapy: A systematic review of self‐report, idiographic instruments. Clinical Psychology: Science and Practice, 26(3), e12281. 10.1111/cpsp.12281

[papt70094-bib-0027] Locke, E. A. (1968). Toward a theory of task motivation and incentives. Organizational Behavior and Human Performance, 3, 157–189. 10.1016/0030-5073(68)90004-4

[papt70094-bib-0028] McMurran, M. , Cox, W. M. , Whitham, D. , & Hedges, L. (2013). The addition of a goal‐based motivational interview to treatment as usual to enhance engagement and reduce dropouts in a personality disorder treatment service: Results of a feasibility study for a randomised controlled trial. Trials, 14, 1–10. 10.1186/1745-6215-14-50 PMC3598789 23414174

[papt70094-bib-0029] Miller, S. D. , Duncan, B. L. , Brown, J. , Sparks, J. A. , & Claud, D. A. (2003). The outcome rating scale: A preliminary study of the reliability, validity, and feasibility of a brief visual analog measure. Journal of Brief Therapy, 2(2), 91–100.

[papt70094-bib-0030] Murray, D. M. , Varnell, S. P. , & Blitstein, J. L. (2004). Design and analysis of group‐randomized trials: A review of recent methodological developments. American Journal of Public Health, 94, 423–432. 10.2105/AJPH.94.3.423 PMC1448268 14998806

[papt70094-bib-0031] NHS England . (2024). Children and Young People's Psychological Trainings (CYP PT).

[papt70094-bib-0033] Redmayne, K. A. , Reese, R. J. , Zetzer, H. A. , Conoley, C. W. , & Owen, J. (2024). Do routine outcome monitoring measures tell the same story? Evaluating outcome trajectories and clinical outcomes for the outcome rating scale and outcome questionnaire – 45. Psychotherapy Research, 34(2), 195–204. 10.1080/10503307.2023.2181113 36931233

[papt70094-bib-0035] Ryan, R. , Berry, K. , & Hartley, S. (2023). Review: Therapist factors and their impact on therapeutic alliance and outcomes in child and adolescent mental health – A systematic review. Child and Adolescent Mental Health, 28(2), 195–211. 10.1111/camh.12518 34763371

[papt70094-bib-0036] Sales, C. , Ashworth, M. , Ayis, S. , Barkham, M. , Edbrooke‐Childs, J. , Faisca, J. , Jacob, J. , Xu, D. , & Cooper, M. (2023). Idiographic patient reported outcome measures (I‐PROMs) for routine outcome monitoring in psychological therapies: A position paper. Journal of Clinical Psychology, 79(3), 596–621. 10.1002/jclp.23319 35194799

[papt70094-bib-0037] Stafford, M. R. , Cooper, M. , Barkham, M. , Beecham, J. , Bower, P. , Cromarty, K. , Fugard, A. J. , Jackson, C. , Pearce, P. , Ryder, R. , & Street, C. (2018). Effectiveness and cost‐effectiveness of humanistic counselling in schools for young people with emotional distress (ETHOS): Study protocol for a randomised controlled trial. Trials, 19(1), 1–16. 10.1186/s13063-018-2538-2 PMC5845203 29523206

[papt70094-bib-0038] Tryon, G. S. (2018). Goals and psychotherapy research. In M. Cooper & D. Law (Eds.), Working with goals in psychotherapy and counselling (pp. 87–109). Oxford University Press. 10.1093/med-psych/9780198793687.003.0005

[papt70094-bib-0039] Twigg, E. , Barkham, M. , Bewick, B. M. , Mulhern, B. , Connell, J. , & Cooper, M. (2009). The young Person's CORE: Development of a brief outcome measure for young people. Counselling and Psychotherapy Research, 9(3), 160–168. 10.1080/14733140902979722

[papt70094-bib-0040] Twigg, E. , Cooper, M. , Evans, C. , Freire, E. , Mellor‐Clark, J. , McInnes, B. , & Barkham, M. (2016). Acceptability, reliability, referential distributions and sensitivity to change in the young Person's clinical outcomes in routine evaluation (YPCORE) outcome measure: Replication and refinement. Child and Adolescent Mental Health, 21(2), 115–123. 10.1111/camh.12128 32680371

